# From Systemic Stress to Ovarian Failure: Heat Stress-Induced Infertility in Pigs as a Model for Reproductive Dysfunction

**DOI:** 10.3390/cimb48030304

**Published:** 2026-03-12

**Authors:** Ramanathan Kasimanickam, Joao C. P. Ferreira, John P. Kastelic

**Affiliations:** 1 College of Veterinary Medicine, Washington State University, Pullman, WA 99164, USA; 2 School of Veterinary Medicine and Animal Science, São Paulo State University-UNESP, Botucatu 18618-81, SP, Brazil; joao.cp.ferreira@unesp.br; 3 Faculty of Veterinary Medicine, University of Calgary, Calgary, AB T2N 4Z6, Canada; jpkastel@ucalgary.ca

**Keywords:** porcine reproduction, heat stress, ovarian function, endocrine disruption, intestinal permeability, follicle development

## Abstract

Heat stress (HS) occurs when animals are unable to effectively dissipate excess body heat, leading to increased core temperature and physiological imbalance. In mammals, HS negatively affects female reproduction. Infertility associated with HS is well documented in swine and is increasingly recognized in other mammals, including humans. HS disrupts several systemic processes that are essential for normal reproductive function, including endocrine regulation, nutrient metabolism, immune activity, and intestinal barrier integrity. Reduced feed intake and changes in metabolic hormones such as insulin and prolactin can impair ovarian function. Increased intestinal permeability during HS may allow bacterial endotoxins to enter the bloodstream, triggering inflammation that further compromises reproductive physiology. At the ovarian level, HS alters key cellular pathways involved in cell survival and metabolism, including Janus Kinase/Signal Transducer and Activator of Transcription (JAK–STAT), Phosphoinositide 3-Kinase/Protein Kinase B (PI3K/AKT), oxidative stress responses, autophagy, apoptosis, and heat shock protein expression. These changes disrupt follicular development, hormone production, oocyte quality, and corpus luteum function, resulting in reduced conception rates and increased embryonic loss. This review summarizes current knowledge of systemic and ovarian mechanisms by which HS impairs female reproduction in *pigs* and identifies areas requiring further investigation to improve fertility under increasing environmental temperatures.

## 1. Introduction

Global environmental temperatures have steadily increased over recent decades, with an average rise of ~0.7 °C per decade, and projections suggesting a cumulative increase of 1–2 °C by 2050 [1,2,3]. This warming trend is accompanied by increased frequency and intensity of extreme weather events, including prolonged heatwaves, which pose challenges to human and animal health [4]. Elevated ambient temperatures are associated with increased incidence of infectious diseases, malnutrition, cardiovascular and respiratory disorders, and even mortality, particularly in vulnerable populations such as the elderly, young, and those with limited socioeconomic resources [5,6,7]. Urban heat islands and limited access to cooling technologies, such as air conditioning, further exacerbate heat-related health risks [8,9]. In this context, physiological effects of heat stress (HS) on mammals have become a major concern, with reproductive dysfunction emerging as a particularly sensitive end point.

In mammals, HS arises when environmental heat load, combined with endogenous metabolic heat production, exceeds the body’s capacity for heat dissipation [10]. Thermoregulatory mechanisms, including increased peripheral blood flow, sweating, and panting, facilitate heat dissipation and maintenance of thermal homeostasis. However, prolonged or severe heat exposure can exceed adaptive capacity, resulting in physiological dysfunction. In production animals such as swine, heat stress is associated with reduced growth performance, decreased lactation efficiency, impaired feed efficiency, and compromised reproductive function [11]. Female reproduction is highly sensitive to HS, with consequences ranging from anovulation and impaired estrous cyclicity to decreased conception rates, early embryonic loss, and pregnancy failure [12,13,14]. In *pigs*, seasonal infertility, most pronounced during summer and early autumn in North America, is widely recognized and reflects the detrimental effects of heat-associated metabolic and energetic challenges on reproductive efficiency [15].

Heat stress (HS) imposes significant challenges to female reproductive physiology, particularly in livestock species such as *pigs* [16]. While various mammalian models have been used to study HS-induced infertility, this review focuses primarily on the porcine model due to its agricultural importance and translational relevance [16,17]. References to other species—including murine and bovine studies—are included selectively to provide mechanistic context and to highlight conserved pathways, but *pigs* remain the central system for examining how heat stress affects ovarian function, folliculogenesis, and endocrine regulation.

The porcine model is especially relevant for studying HS-induced reproductive dysfunction due to its agricultural importance and translational value for human reproductive physiology [18,19]. *Pigs* share several physiological and reproductive similarities with humans, including comparable ovarian architecture, folliculogenesis dynamics, steroidogenic regulation, and endocrine signaling pathways. These similarities make the porcine ovary a valuable system for investigating mechanisms that may extend beyond livestock species. Importantly, many intracellular pathways disrupted by heat stress—including the phosphoinositide 3-kinase/protein kinase B (PI3K/AKT) signaling pathway, Janus kinase/signal transducer and activator of transcription (JAK–STAT) signaling, inflammatory cascades, and oxidative stress–mediated responses—are highly conserved across mammalian systems [20,21]. Consequently, mechanistic insights derived from porcine ovarian responses to heat stress may provide broader biological understanding of how thermal stress perturbs follicular development, oocyte competence, and overall ovarian function. Recognizing these conserved mechanisms strengthens the translational relevance of the porcine model for investigating fundamental pathways underlying heat stress–associated reproductive impairment.

Importantly, HS affects both systemic and ovarian-specific processes, influencing endocrine signaling, nutrient metabolism, immune responses, and cellular stress pathways, each of which can independently or synergistically compromise fertility. At the ovarian level, HS alters key intracellular signaling pathways, including JAK–STAT, PI3K/AKT, toll-like receptor 4 (TLR4)–mediated inflammatory signaling, oxidative stress regulation, and heat shock protein expression [22,23]. These molecular disruptions affect ovarian follicle development, steroid hormone synthesis, and corpus luteum (CL) function, ultimately reducing reproductive success.

This review aims to synthesize current knowledge on how HS affects female reproduction in *pigs*, emphasizing both systemic physiological changes and direct ovarian impacts. We highlight key mechanisms underlying fertility impairment, including hypophagia, intestinal hyperpermeability, endocrine dysregulation, and molecular alterations in the ovary. By focusing on the porcine model, this work provides a detailed mechanistic framework that can inform agricultural management practices and translational research into human reproductive health. Furthermore, it identifies critical gaps in understanding, including temporal dynamics, tissue-specific vulnerabilities, and potential strategies for mitigation, which are essential for developing interventions to preserve fertility under increasingly common HS conditions.

## 2. Heat Stress and Its Multifaceted Disruption of Female Reproduction

HS exerts profound negative effects on female reproductive function across mammalian species, with consequences ranging from altered ovarian activity to impaired pregnancy outcomes. In *pigs*, HS has been associated with anovulation, decreased conception rates, and compromised pregnancy maintenance, highlighting the vulnerability of the female reproductive system to elevated ambient temperatures [12,13,14]. These reproductive impairments are not unique to swine; studies in cattle demonstrate that in vitro exposure of preovulatory follicles to HS decreases estradiol (E2) production in granulosa cells [24,25], reduces oocyte competence [26,27], and ultimately diminishes fertility outcomes in vivo [28,29].

Beyond ovarian follicular development, HS negatively affects embryonic and fetal growth. In ruminants, particularly cattle, exposure to elevated ambient temperatures during early gestation adversely affects embryonic development [30,31] and is associated with restricted fetal growth later in pregnancy [32,33,34]. Similarly, in swine, in utero heat stress has persistent postnatal effects, including elevated core body temperature and altered body composition characterized by increased adiposity, with evidence of disrupted somatic and organ development [35,36]. Comparable outcomes have been reported in ruminants, demonstrating that gestational heat exposure can alter thermoregulation and productivity in postnatal life [37].

In humans, effects of elevated ambient temperatures on female reproductive physiology are increasingly evident. Women exposed to higher temperatures have reduced antral follicle counts, an indicator of diminished ovarian reserve [38], and increased risk of stillbirth [39]. Interestingly, HS tolerance in women is influenced by the menstrual cycle, with improved heat tolerance during the luteal phase, suggesting that sex steroid profiles may modulate thermoregulatory capacity [40]. Pregnancy represents a particularly sensitive window for HS effects; maternal hyperthermia elevates risks of miscarriage [41], hypertension [42], and preterm birth. Analysis of birth outcomes following the 1995 Chicago heat wave revealed a significant association between extreme heat exposure and preterm birth, with disparities across racial groups [43].

**Figure 1:**
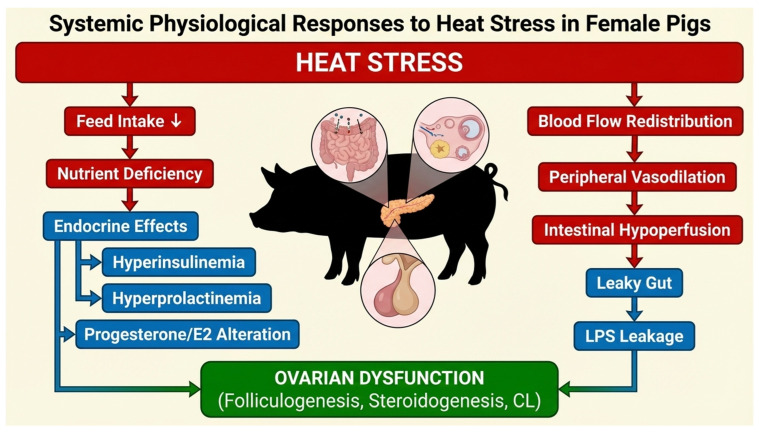
**Systemic physiological responses to heat stress in female** ***pigs***. Schematic diagram illustrating systemic consequences of heat stress (HS) in female *pigs*. HS triggers a reduction in feed intake (inappetence), alterations in endocrine signaling (increased insulin, prolactin, altered progesterone and estradiol), and metabolic disruptions. Blood is redirected to the periphery to enhance heat dissipation, causing intestinal hypoperfusion, hyperpermeability, and low-level endotoxemia [lipopolysaccharide (LPS) leakage]. These systemic alterations independently and synergistically impair ovarian function and reproductive competence. Key organs affected include the gastrointestinal tract, pancreas, pituitary, and ovaries. Arrows indicate causal links between HS, systemic alterations, and reproductive outcomes.

Deleterious effects of HS on female reproduction arise from both direct and indirect mechanisms. Elevated temperatures can directly impair ovarian function and embryonic development; in addition, systemic physiological alterations also contribute to infertility. These systemic effects include reduced nutrient intake, intestinal hyperpermeability (“leaky gut”), systemic inflammation, and endocrine dysregulation, all of which can compromise reproductive competence, even in the absence of overt hyperthermia of reproductive organs (Figure 1). Understanding these interconnected pathways is essential for identifying mitigation strategies and elucidating the multifactorial nature of HS-induced reproductive dysfunction.

HS elicits a variety of systemic physiological alterations, many of which independently compromise reproductive function. These include hypophagia and reduced nutritional intake, intestinal hyperpermeability and endotoxemia, systemic inflammation, and endocrine dysregulation. The subsequent sections elaborate on these mechanisms and their potential contributions to fertility impairment.

### 2.1. Heat Stress, Reduced Feed Intake, and Consequences for Female Reproduction

Heat stress commonly induces inappetence in mammals, including *pigs*, resulting in a reduced plane of nutrition and negative energy balance [11]. This decreased feed intake is an adaptive thermoregulatory response, as digestion of feed generates metabolic heat that further challenges thermal homeostasis. However, the resulting malnutrition has important consequences for reproductive function. In both humans and animals, undernutrition or caloric restriction is well-documented to impair fertility [44,45,46] by altering hypothalamic–pituitary–gonadal signaling and depleting energy reserves necessary for ovarian function. Specifically, insufficient caloric intake can delay puberty, compromise ovarian follicular development, and reduce ovarian reserve, ultimately impairing fertility potential [47,48].

Metabolic hormones act as critical mediators linking nutrition and reproductive physiology. Leptin, secreted by adipose tissue, and ghrelin, produced by the stomach, are key regulators of energy homeostasis and reproductive maturation [49]. Reduced energy intake lowers circulating leptin and alters ghrelin dynamics, delaying puberty and disrupting gonadotropin secretion in both humans and livestock [50,51,52,53,54]. In dairy cattle, for instance, energy restriction during HS can explain up to 50% of reduced lactational performance [55], suggesting a substantial contribution of decreased nutrient intake to the overall physiological burden of HS. Although direct effects of reduced nutrition on fertility during HS are not fully quantified, it is plausible that energy deficits significantly exacerbate HS-induced reproductive dysfunction.

At the metabolic level, HS alters the serum metabolome in pre-pubertal *pigs*, highlighting systemic changes with potential reproductive implications [56]. For example, aminomalonic acid, a biomarker of protein oxidative stress, was reduced, reflecting altered protein metabolism and cellular stress responses. Serum campesterol concentrations were also decreased; campesterol, structurally similar to cholesterol, competes for absorption and can influence steroidogenesis, implying a potential pathway for endocrine disruption during HS [57]. Conversely, hydroxylamine concentrations were elevated under HS, raising concern due to its mutagenic properties [58]. Amino acid profiles in cumulus–oocyte complexes are also altered under thermal stress, potentially impairing oocyte maturation and competence [59,60].

A reduced plane of nutrition under HS not only deprives females of essential energy and metabolites for ovarian and reproductive function but also induces metabolomic and hormonal changes that may amplify infertility. This underscores the importance of considering nutritional status as a critical, yet often overlooked, factor in HS-induced reproductive impairment.

### 2.2. Gut Under Fire: Hidden Threats to Reproduction

Heat Stress imposes profound physiological challenges in mammals, particularly by disrupting vascular homeostasis to prioritize thermoregulation. During HS, blood is shunted from central organs, including the gastrointestinal tract, toward the periphery to facilitate convective and evaporative heat loss. This adaptive circulatory redistribution maintains core temperature but induces hypoperfusion of the intestine, particularly the jejunum and ileum, which are highly metabolically active and oxygen-dependent [61,62,63]. Enterocytes are extremely sensitive to oxygen and nutrient restriction, and even brief ischemia compromises tight junction integrity, mitochondrial function, and epithelial cell viability [64].

The resultant intestinal barrier dysfunction, commonly referred to as “leaky gut,” allows luminal contents, including microbial products, dietary antigens, and partially digested nutrients, to translocate into portal and systemic circulations [65,66,67]. HS-induced hyperpermeability has been consistently demonstrated across species, including *rodents*, *ruminants*, *pigs*, *dogs*, *cats*, and *humans* [68,69], underscoring its evolutionary conservation as a thermoregulatory tradeoff. The Gram-negative bacterial cell wall component lipopolysaccharide (LPS) is particularly notable because its entry into the circulation causes low-grade, chronic metabolic endotoxemia [70,71,72]. Unlike acute infection-induced endotoxemia, heat stress–associated endotoxemia is typically subclinical and may not elicit a febrile response, yet it can markedly disrupt systemic and reproductive physiology [73].

Heat stress disrupts the intestinal barrier, resulting in translocation of microbial products such as LPS, peptidoglycans, and short-chain fatty acids into systemic circulation [68,74,75]. These signals trigger a cascade of inflammatory and endocrine responses that affect distant organs, including the ovary. Collectively, this sequence can be conceptualized as a gut–systemic–ovarian axis, in which intestinal dysregulation under heat stress drives systemic inflammation, hormonal perturbations, and ultimately ovarian dysfunction [76]. Recognition of this axis provides a framework for integrating gastrointestinal, systemic, and ovarian mechanisms within a single conceptual model of HS-induced reproductive impairment.

### 2.3. Silent Signals: How LPS Disrupts Ovarian Function

Building on gut-derived systemic stressors, heat stress also affects metabolic and endocrine parameters, which collectively influence ovarian signaling and follicle survival. LPS activates the immune system primarily through the TLR4 pathway, inducing proinflammatory cytokine production and oxidative stress [77]. Ovarian tissue is directly responsive to systemic endotoxins; bovine ovarian cortical strips enriched in primordial follicles had increased atresia and fewer follicles after LPS exposure in vitro [78]. These effects are *TLR4*-dependent, as *TLR4*-deficient mice resist LPS-induced follicle depletion, confirming that the ovary is a target of circulating microbial products.

Lipopolysaccharide alters reproductive endocrine function by reducing follicular fluid estradiol concentrations [79], suppressing pituitary luteinizing hormone release [80], modulating granulosa cell steroidogenic capacity [81], and increasing ovarian TLR4 protein abundance [73,81]. Chronic low-level LPS exposure in post-pubertal, estrus-synchronized *pigs* increases circulating insulin, elevates LPS-binding protein, and raises serum E2 and glucose concentrations, while simultaneously upregulating ovarian *TLR4* [73,81]. Notably, these systemic and ovarian effects occur without fever, highlighting the subtle yet significant endocrine and reproductive consequences of metabolic endotoxemia.

Although LPS has been widely used as a model for metabolic endotoxemia, intestinal barrier dysfunction during heat stress likely permits translocation of a broader spectrum of microbial and metabolic products. Components derived from Gram-positive bacteria, including peptidoglycans and lipoteichoic acids, can activate innate immune signaling through pattern recognition receptors such as *TLR2*, potentially contributing to inflammatory responses within reproductive tissues [82,83,84]. In addition, microbial metabolites such as short-chain fatty acids—including acetate, propionate, and butyrate—may influence ovarian physiology through systemic metabolic and epigenetic mechanisms. While SCFAs generally support intestinal barrier integrity and metabolic homeostasis under normal conditions, dysregulated concentrations or altered microbial composition during heat stress may modify endocrine signaling and immune responses relevant to ovarian function [85,86]. Other microbial-associated molecules, including bacterial DNA fragments and secondary bile acid metabolites, may also enter circulation during intestinal hyperpermeability and interact with immune and metabolic pathways. Consequently, reproductive effects attributed solely to LPS likely represent only part of a complex network of gut-derived signals influencing ovarian physiology during heat stress [87].

### 2.4. Lifelong Reproductive Risks of Heat Stress and Gut Dysfunction

The reproductive impact of intestinal hyperpermeability varies across species and developmental stages. In prepubertal *pigs*, heat stress alters the serum metabolome, including reductions in aminomalonic acid and campesterol, indicating disruptions in oxidative stress–related metabolism and sterol homeostasis [56]. Amino acid perturbations also occur in cumulus-oocyte complexes under HS, compromising oocyte competence and developmental potential [58,59]. Therefore, immature females are particularly sensitive to HS-induced gut-derived endotoxemia, which may delay puberty onset and compromise ovarian reserve.

In utero exposure to maternal HS reduces placental efficiency and fetal growth, partially through gut barrier-mediated inflammatory mechanisms [36]. Postnatally, offspring exposed to in utero heat stress often exhibit elevated core body temperature, altered metabolic profiles, and changes in body composition [35,88], suggesting that prenatal heat stress can have lasting developmental and physiological consequences.

## 3. Gut-Endocrine Crosstalk in Heat Stress

HS-induced intestinal hyperpermeability intersects with multiple endocrine pathways critical for reproductive function. The preoptic region of the anterior hypothalamus regulates core temperature and integrates estrogenic inputs, influencing both thermoregulation and reproductive cyclicity [89,90,91,92]. Estradiol increases firing of heat-sensitive neurons [93], suggesting that HS-induced endocrine disruption may exacerbate hyperthermia while simultaneously impacting ovarian feedback mechanisms.

### 3.1. Heat Stress-Induced Insulin Effects on Ovarian Function

Reduced feed intake under HS is an adaptive response to lower the thermic cost of digestion, yet paradoxically, circulating insulin is elevated [11,94,95,96,97,98,99,100,101,102,103,104]. Both basal insulin and glucose-stimulated insulin secretion are increased relative to thermal-neutral controls, implying that hyperinsulinemia is independent of caloric intake. Insulin regulates ovarian follicular growth, steroidogenesis, and oocyte energy metabolism [105,106], and chronic hyperinsulinemia is associated with reduced fecundity and fertility [107]. Mechanistically, insulin amplifies ovarian steroidogenic enzyme activity and influences granulosa and theca cell proliferation; however, excessive insulin may also contribute to luteal dysfunction under HS.

### 3.2. Heat Stress-Induced Prolactin Effects on Ovarian Function

Prolactin (PRL), secreted by anterior pituitary lactotrophs [108], is consistently elevated during HS in *pigs*, *cows*, and other mammals [11,109,110,111,112,113]. PRL is involved in thermoregulation, osmotic fluid balance, sweat production, and potentially heat shock protein induction [114,115,116]. Elevated PRL influences ovarian function by stimulating E2 and progesterone (P4) production, regulating luteal immune cell populations, and modulating ovarian follicular development [117,118]. Hyperprolactinemia causes infertility, amenorrhea, and reduced gonadotropin responsiveness [119,120]. Female Prl^−/−^ mice are infertile, with irregular cyclicity, increased luteal cell death, and reduced P4 production [121,122,123]. Prolactin receptor (*PRLR*) gene variants in *pigs* influence thermotolerance, with long and short isoforms modulating JAK/STAT, Mitogen-activated protein kinases (MAPK), and PI3K signaling [124,125,126,127]. Clearly, HS-induced hyperprolactinemia contributes to reproductive compromise.

### 3.3. Luteal Vulnerability and Progesterone Under Heat Stress

The CL is the primary P4 source in *pigs*, essential for conceptus implantation and pregnancy maintenance [128,129,130]. HS reduces CL weight, possibly due to cellular apoptosis or altered luteal composition, while P4 output per unit tissue may paradoxically increase, reflecting compensatory steroidogenesis [131]. LPS and Tumor Necrosis Factor (TNF) α disrupt luteal function and induce prostaglandin F2 (PGF2) α, contributing to early embryonic loss [132,133,134]. However, exogenous P4 restores CL weight and supports pregnancy under HS, offering a potential mitigation strategy.

### 3.4. Gut-Endocrine-Ovarian Axis: A Network Under Heat Stress

**Figure 2:**
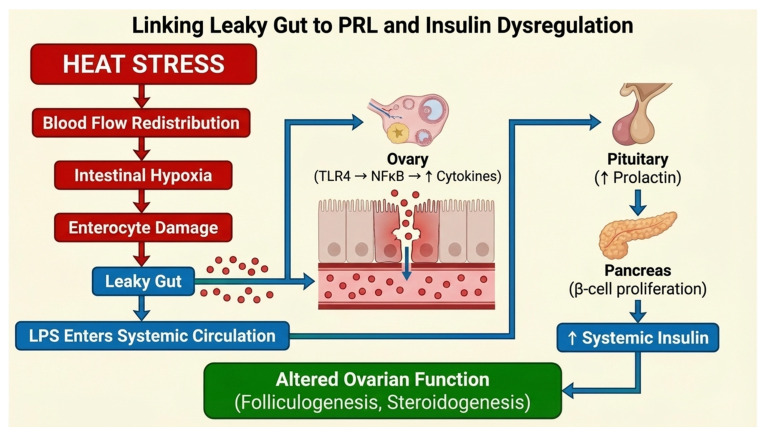
**Proposed link between intestinal hyperpermeability and endocrine/metabolic responses**. Schematic representation of systemic and ovarian responses to heat stress (HS) in *pigs*. Key cytokines, including Tumor Necrosis Factor (*TNF*)-α, interleukin (*IL*)-*1β*, *IL-6*, and *IL-8*, mediate inflammatory signaling that contributes to intestinal barrier dysfunction, systemic endotoxemia, and ovarian perturbations. These systemic changes interact with ovarian signaling pathways—such as phosphoinositide 3-kinase/protein kinase B (PI3K/AKT) signaling pathway, Janus kinase/signal transducer and activator of transcription (JAK–STAT), Toll like receptor (TLR)4-mediated inflammation, oxidative stress, and heat shock protein (HSP) responses—to impair follicular development, steroidogenesis, and corpus luteum function.

Intestinal hyperpermeability, metabolic endotoxemia, insulin hypersecretion, hyperprolactinemia, and CL dysfunction are interlinked. LPS stimulates PRL release [135], PRL promotes pancreatic beta-cell proliferation and insulin secretion [136,137], and extra-pituitary PRL enhances inflammatory signaling [21,138]. PRL also modulates LPS-induced *TLR4*/Nuclear Factor kappa-light-chain-enhancer of activated B cells (NF-κB) signaling [139,140]. Thus, gut-derived endotoxemia is tightly integrated with endocrine and metabolic responses, ultimately influencing ovarian function, follicular development, steroidogenesis, and reproductive success during HS (Figure 2). This proposed sequence linking vascular redistribution, intestinal barrier disruption, endotoxemia, and ovarian molecular responses is conceptually illustrated in Figure 2.

### 3.5. Heat Stress-Induced Oxidative and Inflammatory Challenges to Fertility

Heat stress-induced intestinal hyperpermeability and endotoxemia elevate systemic oxidative stress. Ischemia in enterocytes generates reactive oxygen species (ROS) that can circulate and affect ovarian tissue [64]. ROS impairs oocyte quality, reduces granulosa cell viability, and disrupts steroidogenesis, while proinflammatory cytokines (e.g., *TNFα*, *IL-1β*) induced by LPS further compromise luteal and follicular function [141,142,143,144,145]. Together, oxidative and inflammatory stress represents a central mechanism linking intestinal barrier dysfunction to reduced fertility [142,143,144,145,146].

In summary, HS-induced intestinal hyperpermeability, low-grade endotoxemia, endocrine disruption, and systemic inflammation form a mechanistic network that impairs female reproduction across species. These processes affect ovarian reserve, folliculogenesis, luteal function, steroidogenesis, and metabolic homeostasis, with implications for fertility and pregnancy outcomes. Integrating gut, endocrine, and ovarian responses is critical to understanding and mitigating HS-induced reproductive dysfunction.

A remaining challenge in understanding heat stress-induced reproductive dysfunction is defining the temporal sequence linking systemic physiological disturbances with ovarian molecular responses. Based on currently available evidence, a hypothetical progression can be proposed. In the early phase, elevated environmental temperature triggers thermoregulatory vascular redistribution, increasing peripheral blood flow to facilitate heat dissipation while reducing perfusion of visceral organs, including the gastrointestinal tract. In Stage 1, vascular redistribution likely occurs within hours of heat exposure. Reduced intestinal perfusion then promotes enterocyte hypoxia and epithelial barrier disruption (Stage 2), resulting in increased intestinal permeability (“leaky gut”) [68,84,101]. Subsequent translocation of bacterial products such as lipopolysaccharide into the circulation leads to Stage 3: low-grade metabolic endotoxemia, accompanied by systemic inflammatory signaling, endocrine perturbations, and oxidative stress [147,148]. These systemic alterations ultimately converge on the ovary, where Stage 4 occurs: ovarian molecular remodeling becomes evident through activation of signaling pathways including TLR4/NF-κB, insulin/PI3K/AKT, and JAK–STAT, as well as altered steroidogenesis, heat shock protein expression, and cell death pathways [149,150,151,152,153]. Although the precise timing of these stages likely varies with heat intensity, duration, and physiological state, this conceptual sequence provides a testable framework linking systemic heat stress responses to ovarian dysfunction and may guide future chronobiological investigations of heat stress–induced infertility.

## 4. Heat Stress and the Molecular Machinery of the Ovary

**Figure 3:**
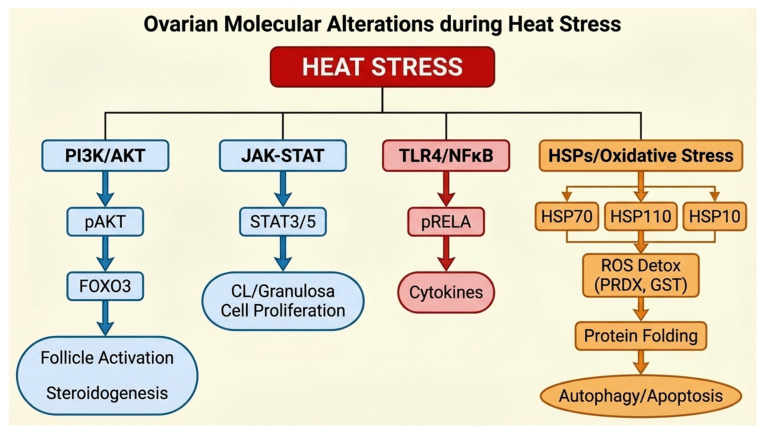
**Ovarian molecular pathways altered by heat stress**. Schematic representation of key ovarian molecular pathways altered by HS in female *pigs*. Heat stress impacts insulin/PI3K/AKT) signaling, JAK–STAT pathways, TLR4/NFκB inflammatory signaling, steroidogenesis (*STAR*, *CYP19A*, P4/E2 production), heat shock protein expression (HSP70, HSP110), oxidative stress responses, and autophagy/cell death mechanisms (*LC3B-II*, *BECN1*, *BCL2* family). These disruptions collectively impair follicle activation, oocyte growth, corpus luteum function, and overall reproductive competence.

HS not only alters systemic physiology but also profoundly affects intracellular signaling within the ovary. These molecular disruptions are linked to hyperinsulinemia, PRL elevation, and metabolic endotoxemia in vivo, ultimately impairing follicle development, steroidogenesis, and oocyte competence. Key pathways affected by HS include insulin/PI3K signaling, LPS/TLR4 inflammatory pathways, steroidogenesis, JAK–STAT signaling, heat shock protein expression, autophagy, apoptosis, and other cell death processes [154]. Collectively, these alterations form a molecular network that underlies reproductive dysfunction in heat-stressed females (Figure 3).

### 4.1. Ovarian Insulin Signaling and PI3K/AKT Pathway Under Heat Stress

The ovary expresses insulin receptors (IRs), heterotetrameric proteins composed of two extracellular α-subunits and two transmembrane β-subunits [73,155,156]. Binding of insulin to the α-subunits activates the β-subunit tyrosine kinase, triggering autophosphorylation and recruitment of insulin receptor substrates (IRS1–4). Disruptions in IRS1 or IRS2 are associated with infertility, highlighting the critical role of insulin signaling in ovarian function [105,157,158]. In postpubertal, estrus-synchronized *pigs*, heat stress during the follicular phase increases ovarian IR abundance, demonstrating that hyperinsulinemia potentiates ovarian insulin signaling [159]. Similarly, prepubertal heat-stressed *pigs* show elevated IRS1 mRNA and phosphorylated IRS1^Tyr632, confirming enhanced insulin-mediated signaling in the ovary [160].

A key downstream effector of insulin signaling is *PI3K*, which regulates primordial follicle survival and activation [20,160,161,162,163,164]. *PI3K* activation leads to phosphorylation of phosphatidylinositol-4,5-bisphosphate to phosphatidylinositol-3,4,5-trisphosphate, recruiting lipid-binding proteins such as *AKT* to the plasma membrane [165,166,167]. *AKT* phosphorylates Forkhead Box O3 (*FOXO3*), a transcription factor that regulates oocyte cell cycle arrest and apoptosis [168,169,170,171]. Phosphorylated *FOXO3* translocates from the nucleus, permitting follicle activation and growth. HS increases ovarian *AKT1* and *FOXO3* mRNA, as well as pAKT1 protein, indicating enhanced PI3K/AKT signaling under heat load [105,157]. However, elevated *FOXO3* activity may inhibit primordial follicle activation, potentially reducing ovarian reserve and impairing fertility [172,173,174].

### 4.2. Ovarian TLR4/NF-κB Signaling in Response to Heat Stress

Heat stress-induced intestinal hyperpermeability facilitates translocation of LPS into the systemic circulation [71,72]. In *pigs* exposed to cyclical HS, ovarian TLR4 protein abundance increases, indicating ovarian responsiveness to endotoxemia [73]. *TLR4* activation triggers a signaling cascade culminating in NFκB activation via phosphorylation of *RELA* and nuclear translocation [175,176]. Activated NFκB upregulates proinflammatory cytokines, including *TNFα* and *IL-1β*, which impair follicle survival and oocyte competence [81,177,178,179].

Heat-stressed post-pubertal females exhibit increased ovarian *pRELA*, supporting the concept that the ovary responds directly to systemic endotoxemia. These findings link HS-induced intestinal barrier dysfunction to local ovarian inflammation and provide a molecular mechanism for follicular atresia observed during heat episodes.

Direct evidence from both in vivo and in vitro studies demonstrates that heat stress modulates PI3K/AKT signaling in ovarian granulosa cells. In vitro experiments using heat-stressed porcine granulosa cells show decreased phosphorylation of *AKT* and downstream targets, correlating with impaired steroidogenesis and increased apoptotic susceptibility [180,181]. Complementary in vivo studies in heat-stressed sows confirm similar disruptions in PI3K/AKT activity, highlighting this pathway’s central role in regulating follicular survival and ovarian function during thermal stress [20,151]. Mechanistically, TLR4/NF-κB-mediated inflammatory signaling can negatively influence PI3K/AKT activity, linking systemic endotoxemia to suppression of pro-survival pathways in ovarian follicles [149]. These findings provide mechanistic support for the contribution of PI3K/AKT pathway alterations to heat stress-induced reproductive dysfunction.

### 4.3. Impact of Heat Stress on Ovarian Steroidogenesis

Ovarian steroidogenesis is acutely sensitive to HS. In granulosa cells from mice, HS reduces E2 and P4 along with decreased expression of steroidogenic enzymes [182]. Conversely, in pre-pubertal heat-stressed *pigs*, Steroidogenic Acute Regulatory protein (*STAR*) and cytochrome P450 family 19 subfamily A member 1 (*CYP19A1*) mRNA are increased, suggesting compensatory attempts to maintain steroid production under heat load [21]. *STAR* mediates cholesterol transport into mitochondria, initiating steroid synthesis, while CYP19A1 catalyzes the final step in E2 production.

Interestingly, in luteal tissue of *pigs* exposed to HS, *STAR*, 3β-hydroxysteroid dehydrogenase, and PGF2α receptor abundance were unchanged despite increased P4 per mg of CL tissue [131], indicating that HS may enhance steroidogenic efficiency to counter reduced luteal mass. These findings emphasize nuanced effects of HS on ovarian steroidogenesis.

### 4.4. Prolactin-Mediated Ovarian JAK–STAT Activation Under Heat Stress

PRL-mediated JAK–STAT signaling is a critical ovarian pathway affected by HS. Heat-stressed pigs exhibit increased *pSTAT5α*/*β^Tyr694*/*699* during the follicular phase and elevated *STAT3* in the luteal phase [183]. These molecular changes reflect systemic hyperprolactinemia and indicate direct modulation of ovarian signaling, potentially impacting granulosa cell proliferation, luteal function, and steroidogenesis.

### 4.5. Heat Shock Proteins in the Ovarian Heat Stress Response

Heat shock proteins (HSPs) act as molecular chaperones, assisting in protein folding, mitochondrial function, and stress response [184,185]. Constitutive HSPs maintain basal protein homeostasis, whereas inducible HSPs respond to HS, oxidative stress, and inflammatory stimuli. In *pigs*, *HSPA1A* (*HSP70*) and *HSPE1* (*HSP10*) increase in response to HS, particularly in granulosa cells and oocytes [186,187]. *HSPH1* has bidirectional regulation, depending on thermal and nutritional conditions [183,188]. These chaperones protect ovarian cells from protein misfolding and mitochondrial dysfunction induced by thermal and oxidative stress.

## 5. Heat Stress-Induced Modulation of Ovarian Cell Death Pathways

HS alters ovarian cell homeostasis by modulating autophagy and apoptosis, critical processes for removing damaged cellular components [189]. Morphological changes such as vacuolization occur in both oocytes and granulosa cells under HS [180]. Furthermore, HS increases protein abundance of Beclin 1 (*BECN1*) and Light Chain 3 Beta (*LC3B*)-II while decreasing *ATG12* in ovarian cells, suggesting a dysregulated autophagic response [181].

Throughout the literature, it is critical to distinguish between in vitro and in vivo findings, as the experimental context can influence observed responses. For example, in vitro studies using isolated porcine granulosa cells [22,190] reveal direct effects of heat stress on steroidogenesis and intracellular signaling, whereas in vivo studies in heat-stressed sows [22,190] capture the integrated response of ovarian tissue under systemic physiological challenges. Notably, many intracellular pathways disrupted by heat stress—including PI3K/AKT, JAK–STAT, TLR4-mediated inflammatory cascades, and oxidative stress–mediated responses—show conserved alterations across these experimental systems, supporting their relevance to reproductive impairment [20,149,151,183].

In prophase I-arrested oocytes, phosphorylated B-cell lymphoma 2 (*BCL2*) and *BCL2L1* are elevated under HS, indicating altered apoptosis regulation [168]. In cultured *pig* oocytes, HS increases the Autophagy-related protein (ATG) 12–ATG5 complex and accelerates LC3B-II turnover, further demonstrating modifications to autophagic machinery [181]. Collectively, these alterations highlight that HS disrupts the balance between autophagy and apoptosis, potentially compromising follicle quality and oocyte survival.

It should be noted that besides autophagy and apoptosis, heat stress affects healthy follicular development through other regulated cell death mechanisms, including necroptosis, pyroptosis, and parthanatos, particularly in response to physiological stressors such as oxidative stress [191,192,193]. Heat stress disrupts follicular health not only by inducing apoptosis and autophagy but also by causing oxidative stress, damaging mitochondrial function, and altering steroidogenesis (significantly reducing estradiol). Together, these mechanisms lead to accelerated follicular atresia, impaired oocyte maturation, and reduced granulosa cell proliferation [191,192,193].

## 6. Proteomic Remodeling and Integrated Ovarian Signaling Networks of the Ovary Under Heat Stress

### 6.1. Proteomic Remodeling

HS induces broad proteomic changes in ovarian tissue. Transcriptional profiling of cumulus–oocyte complexes in *pigs* identified altered mRNAs linked to HSP response and extracellular matrix organization [194]. An untargeted Liquid Chromatography-Tandem Mass Spectrometry (LC–MS/MS) proteomic analysis in pre-pubertal gilts revealed 178 proteins affected by HS, including 76 upregulated and 102 downregulated proteins [184,186,188]. Notably, 79 proteins were consistently altered across multiple thermal and nutritional comparisons.

HS reduces hypoxia upregulated protein 1 (HYOU1), indicating ovarian hypoxia, consistent with reduced blood flow during HS. Proteins related to oxidative stress, peroxiredoxin, thioredoxin-like 1, and glutathione S-transferase, were altered, supporting the notion that HS induces oxidative damage [195,196,197]. Immune-responsive proteins were also affected, including increased complement C2, C8 alpha chain, Galectin-10, heterogeneous nuclear ribonucleoprotein L-like, and decreased Ficolin-2, reflecting immune activation within the ovary [184,186,198].

Proteins involved in chaperoning, extracellular matrix remodeling, metabolism, and signaling were also altered, providing a molecular basis for systemic reproductive consequences observed during HS. HSP family members HSPA1A, HSPE1, and HSPH1 mirrored targeted studies, emphasizing their central role in protecting ovarian function under thermal stress [186,199].

### 6.2. Integrated Ovarian Signaling Networks Under Heat Stress

Collectively, HS orchestrates a multifaceted network of intracellular ovarian signaling disruptions. Enhanced insulin/PI3K/AKT signaling influences follicle activation and oocyte growth, while LPS/TLR4 and JAK–STAT pathways mediate inflammatory and endocrine signals [20,149,151,183]. Heat shock proteins mitigate protein misfolding and mitochondrial stress, while autophagy/apoptosis pathways regulate removal of damaged cellular components. Proteomic changes highlight oxidative stress, immune activation, and extracellular matrix remodeling as additional mechanisms of ovarian dysfunction.

These molecular perturbations, in combination with systemic alterations (hyperinsulinemia, hyperprolactinemia, and endotoxemia), provide a mechanistic framework explaining HS-induced reductions in follicle quality, steroidogenesis, oocyte competence, and early embryonic survival. Across species and developmental stages, these pathways appear highly conserved, underscoring their importance as potential targets for interventions to improve reproductive outcomes under thermal stress.

## 7. Future Directions for Mitigating Heat Stress-Induced Reproductive Dysfunction

HS represents a multifactorial challenge to female fertility, and future research should aim to unravel the temporal and mechanistic sequences of systemic and ovarian responses. Integrity of the intestinal barrier under HS is critical. Although LPS has been widely used to model endotoxemia, the intestine contains a complex milieu of bacterial products and metabolites that may differently influence ovarian signaling. Systematic evaluation of additional microbial and metabolic constituents in heat-stressed females is therefore warranted to better understand the interplay between gut-derived signals and reproductive dysfunction. Complementary to intestinal health, altered blood flow during HS may reduce ovarian delivery of nutrients and hormones, directly impairing follicle development and steroidogenesis [200]. Future studies employing non-invasive imaging or flow-sensitive biomarkers could clarify the extent to which vascular redistribution contributes to reproductive deficits.

Nutritional interventions aimed at sustaining feed intake or supporting metabolic balance during heat episodes may also hold promise; however, these approaches require careful optimization to avoid inducing additional metabolic stress. For example, excessive energy supplementation may overstimulate insulin signaling pathways, leading to hyperinsulinemia and altered metabolic partitioning that could disrupt ovarian endocrine regulation. Similarly, nutritional strategies that strongly stimulate anabolic pathways may alter the dynamics of the insulin-like growth factor (IGF) system, potentially affecting follicular growth, steroidogenesis, and luteal function [21]. Imbalances in these metabolic regulators may exacerbate endocrine dysregulation rather than improve reproductive outcomes. Therefore, nutritional interventions designed to mitigate HS should aim to support metabolic homeostasis while avoiding excessive activation of insulin- or IGF-mediated pathways. Future studies integrating metabolic, endocrine, and reproductive parameters will be essential to define optimal dietary strategies that preserve ovarian function under conditions of thermal stress.

At the molecular level, ovarian responses to HS include insulin/PI3K signaling, LPS/TLR4-mediated inflammation, JAK–STAT signaling, oxidative stress, and autophagy/apoptosis pathways [180,181,183,189]. Understanding the temporal dynamics and tissue-specific interactions among these pathways could reveal intervention windows for pharmacological, nutritional, or environmental mitigation. Importantly, any strategy must balance reproductive preservation with overall animal health, given that the systemic HS response prioritizes survival. Extending these investigations across species and developmental stages will improve translational relevance and may identify conserved mechanisms of HS resilience in female reproduction. Ultimately, integrative studies combining systemic physiology, ovarian molecular biology, and targeted interventions will be critical to reduce HS-induced infertility.

## 8. Conclusions

HS imposes profound effects on female reproductive physiology, resulting in infertility through both systemic and ovarian-specific mechanisms. Systemic alterations, including intestinal barrier dysfunction, hyperinsulinemia, hyperprolactinemia, and vascular redistribution, create a cascade of stressors that impact ovarian signaling pathways. Within the ovary, HS modulates insulin/PI3K signaling, steroidogenesis, LPS/TLR4-mediated inflammation, JAK–STAT activation, oxidative stress, and cell death pathways, including autophagy and apoptosis. Heat shock proteins provide partial protection; however, cumulative molecular disruptions impair follicle survival, oocyte quality, and hormonal balance. Experimental findings in porcine models provide a detailed framework linking systemic and local ovarian responses; however, gaps remain regarding species-specific variations and developmental stage-dependent sensitivity to HS. Potential mitigation strategies, such as improving intestinal integrity, sustaining nutrient intake, modulating endocrine responses, or targeting molecular stress pathways, require rigorous evaluation to ensure they do not compromise overall animal health. Importantly, identifying the temporal sequence of physiological and molecular events is essential to determine the primary drivers of HS-induced infertility and to target interventions effectively. Beyond its importance for livestock productivity, the porcine model provides a valuable translational framework for understanding the broader biological consequences of heat stress on ovarian physiology. Due to similarities between *pigs* and *humans* in ovarian morphology, follicular development patterns, and endocrine regulation, mechanistic insights gained from porcine studies may inform general mammalian reproductive biology. Notably, cellular pathways implicated in heat stress-induced ovarian dysfunction—such as PI3K/AKT signaling, JAK–STAT activation, inflammatory responses, mitochondrial dysfunction, and oxidative stress pathways—are conserved across species. Therefore, studies in *pigs* contribute not only to improving reproductive efficiency in livestock but also to advancing our understanding of conserved mechanisms through which environmental stressors impair ovarian function. Overall, the current body of evidence highlights that reproductive dysfunction under HS is multifactorial, arising from the integration of systemic, cellular, and molecular perturbations. Future work focused on bridging these scales, linking systemic physiological responses with ovarian-specific molecular pathways, will be critical for developing targeted strategies to preserve fertility in heat-stressed females and may also provide insights relevant across mammalian species.

## Data Availability

No new data were created or analyzed in this study. Data sharing is not applicable to this article.
